# Frontalis Sling for the Treatment of Congenital Ptosis

**Published:** 2016-04-04

**Authors:** Krishna S. Vyas, Urian Kim, William D. North, Daniel Stewart

**Affiliations:** Division of Plastic and Reconstructive Surgery, Department of Surgery, University of Kentucky College of Medicine, Lexington

**Keywords:** ptosis, frontalis sling, fascia lata, levator palpebrae superioris, blepharoptosis

## DESCRIPTION

A 28-year-old man with drooping right eyelid since birth was concerned about visual field defects and his appearance. He had no medical or surgical history.

## QUESTIONS

1. **What are the relevant anatomical structures for the elevation of the upper eyelid?**
2. **What are the common congenital and acquired causes of ptosis?**
3. **How is ptosis surgically treated, and what are the technical details?**
4. **What are the surgical techniques of the frontalis sling procedure?**

## DISCUSSION

Three muscles are involved in the elevation of the upper eyelid. The levator palpebrae superioris is the muscle in the orbit that elevates the upper eyelid 10 to 12 mm and is innervated by the third cranial nerve. This muscle becomes a tendinous aponeurosis that fuses with the superior tarsal plate and the dermis of the upper eyelid. Muller's muscle is an accessory smooth muscle innervated by the sympathetic nervous system that arises under the levator palpebrae superioris to attach to the superior tarsus. Muller's muscle elevates the upper eyelid an additional 2 to 3 mm. The frontalis muscle, innervated by cranial nerve VII, elevates the brow along with the upper eyelid. This muscle is attached to the skin of the eyebrows, which joins the galea aponeurotica below the coronal suture.

Eyelid ptosis is drooping of the upper eyelid to a position that is lower than normal due to a congenital or acquired abnormality of the motor system that elevates the eyelid. Ptosis can result from a lesion at any point along the pathway between the cerebral cortex and the levator muscle. Congenital ptosis often results from myogenic dysgenesis of the levator palpebrae superioris.^1^ In such cases, the levator muscle and aponeurosis tissue are replaced by fat and fibrous tissue, reducing the ability to contract or relax. However, a variety of genetic or chromosomal defects and neurological dysfunctions, such as blepharophimosis syndrome and Horner's syndrome, can also result in ptosis. Acquired ptosis is most often aponeurotic, which can occur as a result of dehiscence, stretching, disinsertion, or senescence of the levator aponeurosis. Other causes of acquired ptosis can be neurogenic, such as a third cranial nerve palsy and myasthenia gravis. Trauma, including lacerations, can also result in ptosis. Obstructions of upper lid elevation by neoplasms or postinflammatory scarring are causes for mechanical ptosis.

Management of ptosis is primarily operative, which can improve field of vision and aesthetic appearance. Surgical correction is especially important for young patients with congenital ptosis to prevent the possible development of amblyopia. The surgical procedures depend on the degree of levator function and severity of ptosis.^2^ Mild ptosis of 2 to 3 mm is associated with good levator function of 10 to 15 mm. Moderate ptosis of 3 to 5 mm is associated with fair levator function of 6 to 9 mm. Mild to moderate ptosis with good or fair levator function is most commonly treated with procedures such as tarsal-conjunctival mullerectomy, levator plication, or advancement. Severe ptosis of more than 5 mm with poor levator function of less than 5 mm is treated with a frontalis sling such as silicone or, as reported here, a strip of fascia lata.^3^ The procedure was performed under general anesthesia. The patient's face and right thigh were prepped and draped. Initially the supratarsal fold of the normal left upper eyelid was marked at the mid-pupil level. This was found at 7 mm from the lash line as measured with a caliper. A corresponding point was then marked on the right upper eyelid. This point, representing the level of the new supratarsal fold, was then incorporated into the markings for a conservative blepharoplasty. An epinephrine containing local anesthetic was injected for hemostasis and post-operative analgesia. The blepharoplasty incision was sharply incised. The outlined skin followed by a strip of orbicularis oculi were excised to expose the levator aponeurosis and tarsal plate. An incision centered on the mid-pupillary level was then made just above the eyebrow with exposure of the frontalis muscle. Fascia lata was harvested from the right thigh and provided a 10 cm fascial graft strip approximately 3 mm wide. The fascial graft was then secured to the tarsus of the right upper eyelid at the mid-pupillary line, just medial to the medial limbus and just lateral to the lateral limbus using 5-0 Ethibond (Ethicon, Inc., Somerville, New Jersey) suture. The deep surface of the eyelid was examined to assure that the sutures did not penetrate the full thickness of the tarsal plate and conjunctiva. An empty needle with an eyelet was then used to pass the medial and lateral ends of the graft deep to the orbicularis muscle into the brow incision in pentagonal fashion as described by McCord and Codner.^4^ Tension was placed on the fascial strip and the lid margin was elevated to approximately 1 mm below the upper limbus. The fascia lata strip was then sutured to itself and to the frontalis muscle with 4-0 Ethibond (Ethicon, Inc., Somerville, New Jersey). The eyelid incision was closed with interrupted 5-0 plain gut sutures in an anchor blepharoplasty fashion including levator aponeurosis centrally to assure a crisp supratarsal fold. The brow incision was closed with 5-0 Vicryl (Ethicon, Inc., Somerville, New Jersey) suture to approximately the deep dermis and 5-0 plain gut sutures to approximate the skin edges. On reversal of anesthesia, good symmetry of the upper eyelids was noted.

This patient presents with severe congenital right upper eyelid ptosis with very poor to no levator function and was treated with a frontalis sling using a strip of fascia lata harvested from his right thigh. Treatment with manipulation of the levator muscle and tendon, such as a levator plication or advancement, would not be effective because of his near-absent levator function. The frontalis sling procedure transfers the elevating function of the frontalis muscle to the ptotic eyelid. This can be done by creating a sling from the frontalis muscle to the eyelid, directly suspending the eyelid to the brow. Fascia lata, deep fascia of the thigh, can be used as the sling. Fascia lata is considered one of the best materials for the sling due to its great tensile strength and its ability to biointegrate into the eyelid environment with low rates of granuloma formation, infection, and extrusion. The sling can also be synthetic material such as Supramid® (S. Jackson, Inc., Alexandria, Virginia) suture, Mersilene mesh (Ethicon, Inc., Somerville, New Jersey), or silicone. Silicone slings may be more appropriate for children since they do not scar as much and can be adjusted at a later age.^5^

## Figures and Tables

**Figure 1:**
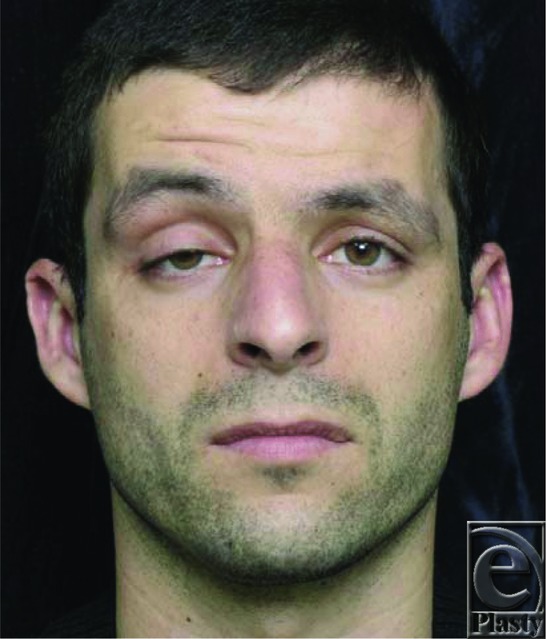
Preoperative photograph demonstrating severe ptosis on the right lid with levator function less than 5 mm (measured by blocking the action of the frontalis muscle). The patient is contracting his right frontalis muscle to keep his right eyelid open. Normal levator function is 11 mm.

**Figure 2:**
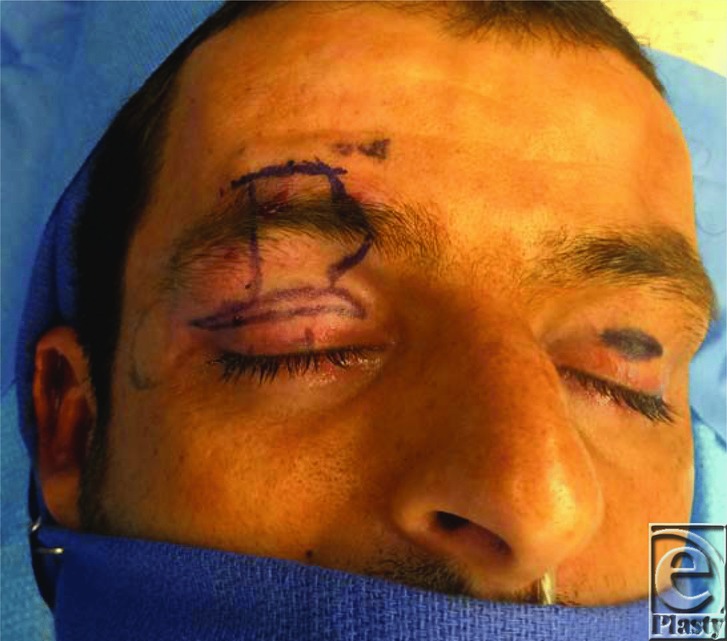
Photograph demonstrating suspension of the upper lid to the frontalis muscle with a strip of fascia lata. A blepharoplasty incision through the supratarsal crease is used to access the levator aponeurosis and tarsal plate.

**Figure 3:**
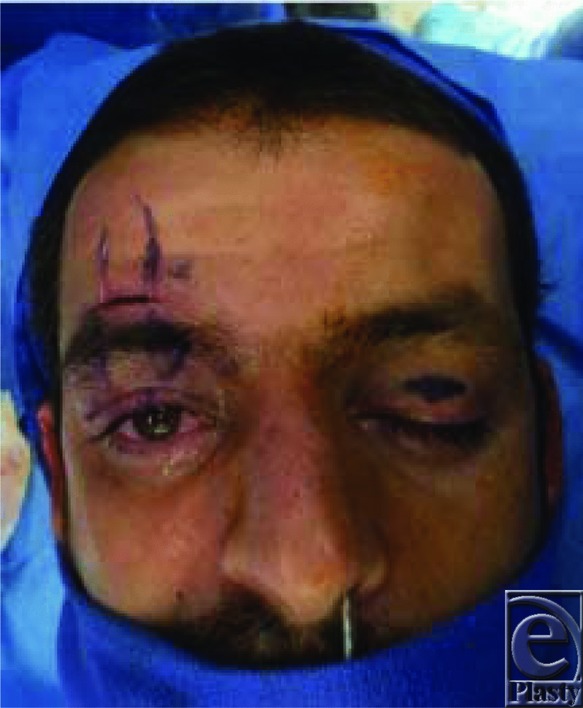
After suturing the fascia lata to the tarsal plate, it was tunneled and sutured to the frontalis muscle.

**Figure 4:**
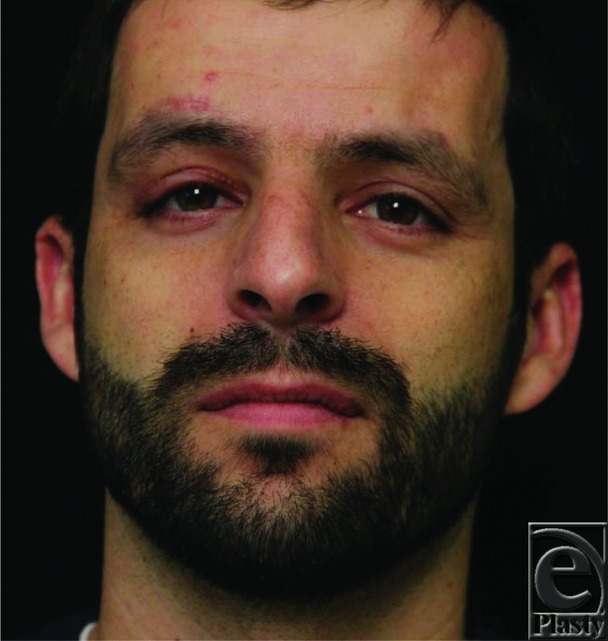
Postoperative photograph at 5 months demonstrating improved margin reflex distance 1 (MDR1).
